# Surgical Management of a Retained Appendicolith Following Laparoscopic Appendectomy

**DOI:** 10.7759/cureus.34701

**Published:** 2023-02-06

**Authors:** Maianh Tran, Dominic Najjar, John Popovich

**Affiliations:** 1 General Surgery, Mercy Medical Center, Des Moines, USA; 2 General Surgery, Des Moines University College of Osteopathic Medicine, Des Moines, USA

**Keywords:** fecalith, retroperitoneal abscess, acute appendicitis, dropped appendicolith, retained appendicolith, laparoscopy appendectomy

## Abstract

Appendicitis is one of the most common surgical emergencies, and surgical intervention remains the gold standard for curative treatment. Although laparoscopic appendectomy is associated with less pain, shorter hospital stays, and earlier mobilization, it is also more frequently complicated by postoperative abscesses. Retained appendicoliths are a rare complication that can serve as a nidus for repeated infections. Laparoscopic removal of the stone can provide definitive source control and reduce repeated hospital admissions. There are many surgical approaches for retrieval and this case, in particular, describes a laparoscopic hand-assisted approach while simultaneously using an interventional radiologic drain to assist with localization.

## Introduction

A retained appendicolith is a rare occurrence with rates of less than 1% [1,2]. Case reports have been discussed with a dropped appendicolith resulting in small bowel obstructions, fistulous tracts, delayed wound closures, and recurrent abscesses [3,4]. The retained stone serves as a nidus for infection, and surgery is the mainstay of treatment for source control. Many approaches exist for stone retrieval, with benefits to each method. With an exploratory laparotomy incision, full exposure, improved visualization, and tactile sensation are allowed. On the other hand, laparoscopy permits minimally invasive incisions, decreased pain, increased recovery time, and fewer wound complications. In addition to these varying surgical approaches, this case report displays the utility of an additional localization technique - maintaining an interventional radiology drain in place to assist with the identification of a retained appendicolith.

## Case presentation

The patient is a 30-year-old male who underwent a laparoscopic appendectomy for necrotic perforated appendicitis with purulent peritonitis in 2017. Following his operation, the patient had a pelvic abscess a month after his index surgery. Given its size of 5 cm and accessibility, interventional radiology was able to place a transgluteal drain. His postoperative course was otherwise uneventful following this until five years later when he returned with a right psoas abscess.

He presented with a month-long history of intermittent constipation and acute right lower quadrant pain for three days. Even with evaluation by urgent care and passage of bowel movements, the pain recurred. Upon evaluation in the emergency department, the patient was found to be tachycardic with a leukocytosis of 11,400/mm^3^. Computed tomography imaging revealed a 2.6 x 2.5 cm abscess in the right lower quadrant extending into the right psoas muscle with focal calcification, representing a retained appendicolith. He was treated with antibiotics, and the abscess was drained by interventional radiology. The following week, the drain was removed after a negative fistulogram.

**Figure 1:**
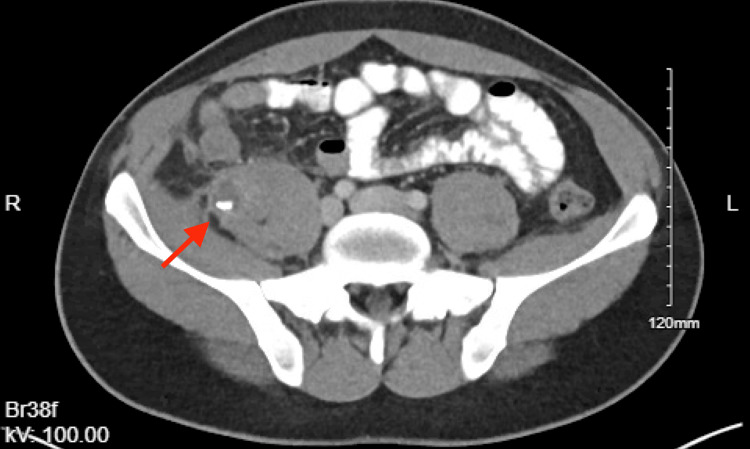
Axial View of Right Psoas Abscess With Retained Appendicolith

**Figure 2:**
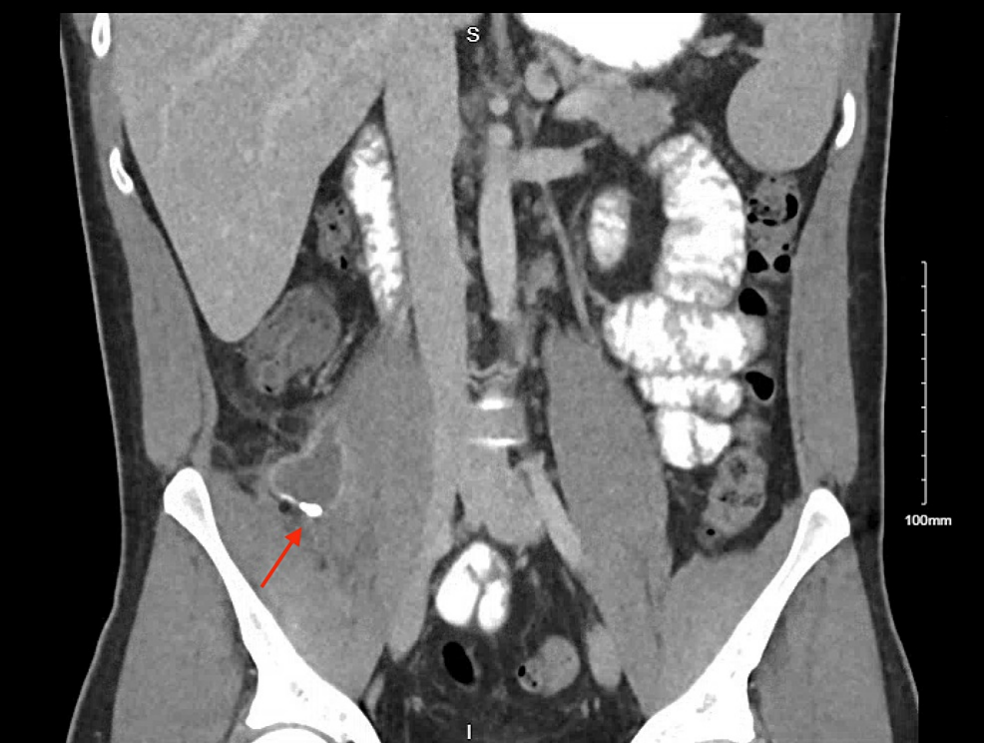
Coronal View of Right Psoas Abscess With Retained Appendicolith

Five months later, the patient presented again with six days of abdominal pain in the right lower quadrant. Although he did not have leukocytosis, the patient was tachycardic and focally tender, and imaging showed a 3.9 x 1.9 x 3.3 cm abscess in the right psoas muscle (Figure 1, Figure 2). He was treated with hospital admission, intravenous antibiotics, and drainage by interventional radiology.

**Figure 3:**
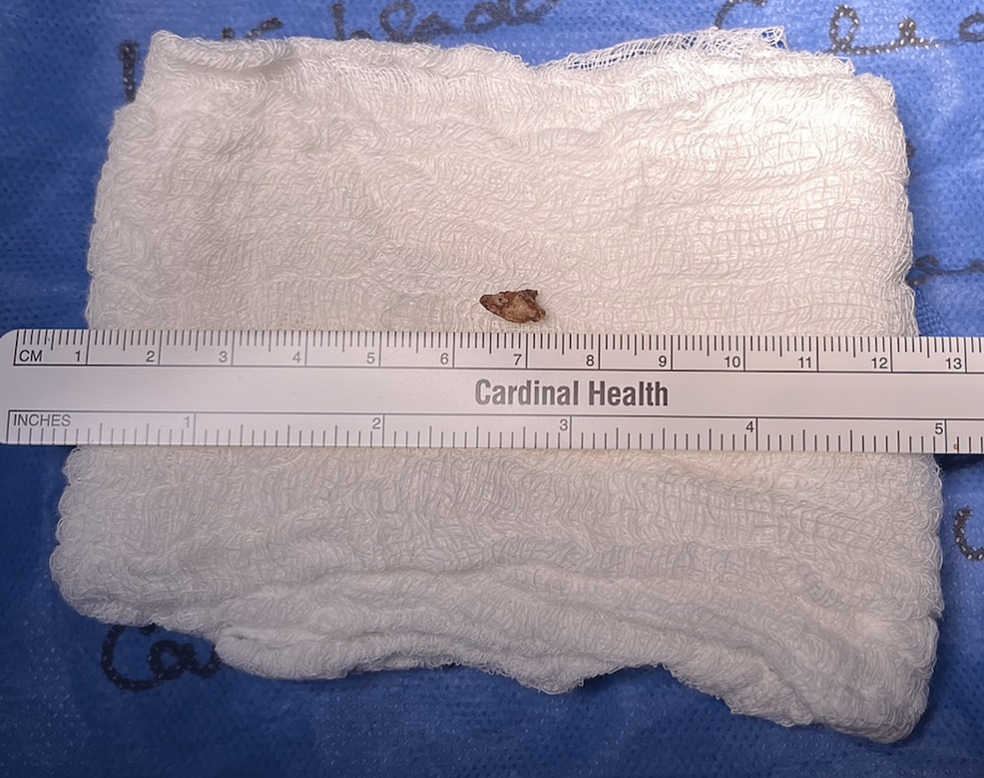
Retrieved Retained Appendicolith

The decision was made to intervene surgically given the recurrent abscesses, prolonged symptoms, and repeated hospitalizations. The drain placed by radiology remained in place for the operation and bilateral ureteral stents were placed by urology given the proximity of the appendicolith to the right ureter. A hand-port-assisted laparoscopic entry was utilized. The retained appendicolith was successfully retrieved (Figure 3) and the pathology was consistent with a fecalith. A surgical drain was left in place, as the radiologic drain was removed intra-operatively. The output initially consisted of 100-300 cc of serosanguinous drainage daily for the first three days. The patient was discharged on postoperative day 3 and with a 10-day course of oral antibiotics. He was seen in the clinic two weeks following the operation and was noted to be doing well with improved pain, fair diet tolerance, and free of fevers. Given the minimal serosanguinous output from the drain, it was discontinued, and he was instructed to follow up only as needed. 

## Discussion

Acute appendicitis is a common cause of abdominal pain that is curative, with a laparoscopic appendectomy. While laparoscopic appendectomy has been shown to have advantages over an open approach, it is also associated with an increased rate of postoperative abscess formation [5]. A retained appendicolith is a rare complication that can lead to postoperative intra-abdominal abscess formation, small bowel obstructions, and fistulous changes [2-4]. Reports have also noted an increased incidence of retained appendicoliths following laparoscopy when compared to open appendectomy [6].

Previous case reports have described different approaches to the management of retained appendicoliths with varying degrees of invasiveness given the contextual factors of each case. There have been successful outcomes in two patients with retained appendicoliths via conservative management involving percutaneous drainage of abscesses and antibiotics [7]. Other cases describe the retrieval of a dropped appendicolith via a percutaneous approach [8] or even with an exploratory laparotomy due to a small bowel obstruction [4].

Re-entry laparoscopy has been reported as an efficacious means of addressing postoperative complications following appendectomy [9]. In this case, percutaneous drainage was unsuccessful. The recurrent nature reinforced the necessity for source control by the removal of the appendicolith. Utilizing laparoscopy and a hand-assisted port allowed for minimally invasive surgery while permitting tactile feedback to manually retrieve the fecalith. In addition, the radiologically placed drain assisted with localization given the location within the psoas muscle. This method avoided a more invasive open surgical approach while still allowing for multiple methods of successful retrieval.

## Conclusions

A retained appendicolith is a nidus for infection and can cause delayed complications following laparoscopic appendectomy. Nonoperative management can lead to recurrent abscesses when the retained appendicolith remains the source of infection. Surgical intervention ultimately provides definitive source control. One surgical approach would be to leave an interventional radiology drain in place to localize the abscess during a hand-assisted laparoscopic retrieval of the stone in the retroperitoneal space.

## References

[1] Ferris M, Quan S, Kaplan BS (2017). The global incidence of appendicitis: a systematic review of population-based studies. Ann Surg.

[2] Horst M, Eich G, Sacher P (2001). Postappendectomy abscess--the role of fecoliths [Article in German]. Swiss Surg.

[3] Ansari FA, Bilal MI, Gondal MU, Latif M, Iqbal N (2021). Delayed presentation of a retained fecalith. Cureus.

[4] Sagkriotis I, Habib Z, Zardab M (2020). Between a rock and a hard place: retained appendicolith causing a mechanical small bowel obstruction. J Surg Case Rep.

[5] Jaschinski T, Mosch CG, Eikermann M, Neugebauer EA, Sauerland S (2018). Laparoscopic versus open surgery for suspected appendicitis. Cochrane Database Syst Rev.

[6] Singh AK, Hahn PF, Gervais D, Vijayraghavan G, Mueller PR (2008). Dropped appendicolith: CT findings and implications for management. AJR Am J Roentgenol.

[7] Albdah A, Aljomah N, Shalhoub M, Zekry A, Beyari N, Bahgat F, AlSubaie N (2021). Benefits of conservative management of a retained appendicolith after laparoscopic appendectomy: a case series. Int J Surg Case Rep.

[8] Hegarty C, Heaslip I, Murphy M, McDermott EW, Brophy DP (2012). Percutaneous removal of a dropped appendicolith using a basket retrieval device and concomitant abscess drainage. J Vasc Interv Radiol.

[9] Casas MA, Laxague F, Schlottmann F, Sadava EE (2021). Re-laparoscopy for the treatment of complications after laparoscopic appendectomy: is it possible to maintain the minimally invasive approach?. Updates Surg.

